# Supplementary material for: Are carbon emissions trading and green financial instruments synergistic? -Comprehensive quantitative research based on content analysis
Source: PLoS One. 2024 Mar 7;19(3):e0298601. doi: 10.1371/journal.pone.0298601 (PMC10919601; doi:10.1371/journal.pone.0298601)
Supplement: S1 Data — (ZIP) [file pone.0298601.s001.zip › Data/地级市Z_F工作报告.zip!/╣π╬≈/╬α╓▌╩╨/└┤╫╘╣1⁄2╓┌║┼í╛┬φ┐╦╩2╛▌═°í┐_2005.docx]

各位代表：

现在，我代表市人民政府向大会报告工作，请予审议，并请市政协各位委员和其他同志提出意见。

一、2004年工作回顾

2004年是我市经济发展和社会事业取得显著成绩的一年。市人民政府在市委的正确领导下，在市人大常委会的监督和市政协的支持下，认真贯彻落实中央和自治区的各项方针政策，以科学发展观统揽全局，全面深入实施开放带动和项目带动战略，制定和出台了一系列强有力的政策措施，通过广大干部群众的共同努力，克服了禽流感影响、煤电油紧张、干旱等困难，完成或超额完成了年初确定的经济和社会发展目标。据统计，2004年全市实现生产总值195.54亿元，同比增长14.1％。其中，第一产业增加值46.67亿元，增长9.3％；第二产业增加值75.93亿元，增长24.8％；第三产业增加值72.94亿元，增长8.1％。规模以上工业增加值28.14亿元，增长28.8％。全市财政收入17.6亿元（不含基金），增长23.7％。全社会固定资产投资73亿元，增长75.2％，全市外贸出口总额2.05亿美元，增长33.7％；实际利用外资9349万美元，增长12.6％；引进市外境内资金33.5亿元，增长363％。全社会消费品零售总额71.31亿元，增长15.1％。城镇居民人均可支配收入6785元；农民人均纯收入2292元，增长14.2％。

总之，2004年全市经济保持了较快增长的态势，超额完成了自治区下达的经济发展“三突破”目标任务，国民经济运行处在10年来最好的时期。全市主要经济指标比上年均有大幅度增长，大部分指标的增长速度是我市多年以来，有的甚至是改革开放以来所没有的。去年，我们主要抓了几方面的工作：

（一）加大农村工作力度，促进农村经济发展和农民增收

为加强农业和农村工作，加快县域经济发展，确保农民增收，我市实施了企业联动、专业村带动、销售队伍拉动、劳务输出驱动、基础设施建设推动的五大措施，并取得显著成效。2004年，全市完成并通过验收的五大措施项目267个，占项目总数98.5％。其中，企业联动和专业村带动农户新增农产品生产基地面积15万亩；销售队伍拉动方面，累计实现销售收入27528万元，完成计划的108.2％；培训农民工10.01万人，输出农民工10.06万人，超额完成了计划任务；基础设施建设共投入资金2.2亿元，其中用于乡村公路建设资金1.88亿元，完成新建、改建乡村公路1265公里，为我市历年乡村公路建设投资最大、项目最多、线路最长的一年。同时还完成除险加固水库16座、渠道硬化防渗33.5公里，新建或修复人饮工程13处，解决了8800多人的饮水问题，新建沼气池9776座。在五大措施的强力推动下，农村经济发展较快，农业结构进一步优化。全市粮食总产量达87.8万吨，优质稻种植面积保持在80％以上；全年实现农业总产值79.6亿元，增长9.8％，是5年来的最好水平，其中水产畜牧业产值完成32.66亿元，占农业总产值的41.04％，比上年增长10.2％；农民人均纯收入增长创8年来最好水平。五大措施的实施得到了自治区领导和国家有关部门的充分肯定和高度评价。

认真贯彻落实党在农村的各项方针政策，巩固税费改革成果，抓好政策性减负措施，去年农民人均税负比税改前减少60.03元。抓好畜禽防疫工作，全市没有发生重大疫情。植树造林、森林防火、防汛抗旱以及扶贫开发等也取得了较好成绩。

（二）加快推进国有企业改革，努力提高工业对经济增长的贡献率

以产权制度改革为重点，突出抓好国有企业的改制工作，先后完成了梧起公司、梧锅公司等42家国有企业的改制，另有21家国有企业正在实施改制，实现了年初确定的国有经济占全市经济总量20％以下的改制目标。加强工业载体建设，市工业园区完成征地3060亩，建成厂房面积达6万平方米，共引进项目20个，其中超亿元的项目8个，已建成投产的项目2个。加大工业招商力度，促进工业投资增长。全年工业项目投资完成20.7亿元，增长124％。其中工业基建项目完成投资14.1亿元，增长227％；技改项目完成投资6.6亿元，增长33％；18个重点项目已有15个开工建设。加大新产品开发力度，主要新产品产值5.2亿元，占主要工业产品产值的21.5％。加强经济运行的协调管理，缓解煤电油运对工业生产的制约。出台了《关于进一步加快工业发展的意见》，建立加快工业发展的激励机制，为加快我市五大工业产业发展提供了强有力的支撑。2004年，年销售收入超亿元的企业共21家，其中超5亿元的企业2家。全市工业对经济增长的贡献率达40.3％，比上年提高8.6个百分点，规模以上工业实现税收4.31亿元，盈亏相抵后实现利润1.53亿元，为历史最好水平。

（三）继续开展三百项目大会战，促进经济提速发展

结合实际贯彻国家宏观调控政策，加大资金筹措力度，坚定不移地推进三百项目大会战。一方面通过置换国有资产，盘活城市资产存量，调整项目投资结构，向商业银行申请短期贷款等办法，千方百计筹措资金，支持项目建设，使政府投资项目基本能按时开工或竣工。另一方面，抓好政府投资项目评审工作，合理控制项目投资规模，大幅度节省了财政资金。同时，扎实推进项目的前期工作，认真抓好招商项目。三百项目大会战取得显著成绩，固定资产投资增幅创1993年以来最好水平。据统计，100个建设项目开工率达到了97％，完成投资额36.01亿元，占年计划的84.37％。100个前期工作项目按时完成目标任务的94个，其中开工建设的23项。100个招商项目中，已签订合同的96个，合同投资总额100.2亿元，合同利用外资64.86亿元，其中56家已开工或投产，当年实际到位资金8.5亿元。

重大项目建设取得了历史性的进展。去年12月，历经全市人民十二年锲而不舍的努力，洛湛铁路广西段在我市举行了隆重的开工动员大会，梧州没有铁路的历史将被改写；经过全市上下的共同努力，长洲水利枢纽建设克服了重重困难成功实现外江截流。苍郁高速公路基本建成；岑梧高速公路建设进展顺利，已完成投资6.38亿元，实现了年度目标。这些重大项目的开工和大力推进，极大地鼓舞了广大干部群众的斗志，为全市经济和社会的快速发展奠定了良好的基础。

（四）加快城市建设步伐，城市面貌发生了较大的变化

克服各种困难，充分调动各方力量，吸纳各类资金，加大城市建设的投入，有效地推进“三年大变”的项目建设。骑楼城改造、地质灾害整治、鸳江丽港和拆迁户安置小区建设、西堤路和竹湾路拓宽、河东主要街道立面装饰等25个工程已经完工或完成年度目标任务，特别是改造后的骑楼城成为梧州一道亮丽的风景线。作为市政建设“一号工程”的地质灾害整治也取得了阶段性成果，共完成投资4220万元，完成了叶琪山专项治理，搬迁高危房屋310户。去年市政建设总投入3.5亿元，新建或改造城市道路11.3公里，改造渠道20.2公里，改造街道立面23万平方米，建设商贸、公共场所等公共设施117万平方米，建成拆迁安置小区3个，住房1100多套，总建筑面积达11万平方米。2004年是旧城改造力度最大、效果最明显，城市面貌发生变化最大的一年，城市建设“两年中变”目标已基本实现。在抓好城市建设的同时，市政府注意加强城市管理工作，改革环卫管理机制，加大对“脏、乱、差”的治理力度，市容市貌明显改善。现在市区的路平了，灯亮了，渠通了，街洁了，城市的品位得到进一步提高。

（五）积极参与区域合作，努力引进外资和扩大出口

进一步加大对外开放的力度，积极融入珠三角经济圈，与江门、中山、肇庆等市建立了经贸合作关系，双方党政代表团进行了互访。落实各项优惠政策，初步解决了我市土地开发成本较高等问题，投资软环境有了较大的改善。出台了《关于强化各级领导干部招商引资目标责任的通知》，改进和完善招商引资工作机制和激励机制，强化招商引资目标任务考核，充分调动各级领导干部招商引资的积极性，招商引资工作取得了改革开放以来最好的成绩。全市新批三资企业65家，合同投资总额2.49亿美元，其中合同外资额1.36亿元，实际到位资金5303万元，增长75.5％。加大外贸出口的扶持力度，克服退税政策调整等因素的影响，加强协调和服务工作，使全市外贸出口主要指标创历史最好水平。特别是机电产品出口增长较快，完成出口额9560万美元，增长35.8％，为全市出口总额的46.6％；高新技术产品出口1746万美元，增长381.3％。

成功举办了首届梧州国际宝石节。加强与东盟各国的经贸合作，与泰国庄他武里府结为友好城市。宝石节期间共签订合同45个，合同投资总额21.81亿元，其中合同外资额6001万美元。

（六）积极组织财政收入，加大财政对经济的支持力度

财政工作做到一手抓组织收入，一手抓支出的调整优化，提高财政资金使用效益。加大对工业和农业经济扶持，特别是支柱财源、基础财源企业的扶持力度，发展壮大原有财源，培育新财源。支持农业五大措施投入资金1亿多元，支持国有企业改制投入2.4亿元，用于工业企业奖励和补助1200多万元，是历年来财政支持经济建设力度最大的一年。加大项目建设投入，财政当年筹措三百项目建设资金10亿元，其中财政直接拨付2.95亿元。加强对财政投资项目的评审工作，合理降低项目成本，为财政核减支出1.8亿元。加大税收征管力度，完善征管办法，推广使用税控机，遏制税源流失，培育和新增纳税超10万元的企业228家，财政收入增幅创1995年以来最好水平。盘活国有资产存量，组织国有资产收入2.9亿元。继续深化部门预算和“收支两条线”管理改革，推进政府集中核算支付和国库集中收付制度改革，加强预算内外资金的统一管理，进一步扩大政府采购范围和规模。同时，市本级还克服财政遇到的各种困难，确保了职工工资和生活补贴的发放及机关的正常运转。

（七）统筹安排，推动各项社会事业协调发展

各项科技创新任务全面完成，第二轮科技创新工作成绩显著，科技特派员试点工作取得初步成效。成功地承办了两广科普大行动暨“西江走廊”科技协作交流活动，增强了两广科普的合作和交流。完成农村中小学校危房改造8.82万平方米，实现年度目标任务，教育质量不断提高。西大梧州分校申本工作稳步推进，市十五中建成并于去年秋季招生。市第三人民医院建成投入使用，医疗救助和疾病预防控制体系不断完善。新型农村合作医疗试点工作取得新成效。文化事业进一步发展，城乡群众文化生活日趋繁荣，市歌舞团赴法国参加华人华侨庆祝我国建国55周年演出取得良好效果。成功举办市第九届运动会，群众体育和竞技体育进一步发展。市广播电视中心建成并投入使用，广播电视宣传和传输播出工作跃上新台阶。出台了《关于加快旅游产业发展的意见》，为加快我市旅游产业发展奠定了基础。深入开展双拥活动，第五次荣获自治区双拥模范城称号。人口自然增长率为7.04‰，控制在预期目标以内。加强反腐倡廉工作，政风行风有明显好转。认真贯彻落实行政许可法，政府依法行政能力得到提高。环境保护、老龄、妇女儿童和残疾人等各项事业稳步发展，人事、民政、机构编制、审计、统计、外事、侨务、信访、档案、修志、新闻出版、地震、人防、民族宗教以及国防教育、民兵预备役建设等各项工作也取得了新的成绩。

（八）抓好社会稳定和安全生产工作

切实巩固“两个确保”工作，全市基本养老金、失业保险金按时足额发放。城市居民最低生活保障制度进一步落实，共有32150人得到低保救助，基本实现了应保尽保。乡镇敬老院等社会福利事业进一步发展，五保村建设取得明显成效。就业和再就业形势良好，全市新增就业16080人，城镇登记失业率为4.13％，实现了全年预期目标，市劳动和社会保障局荣获全国再就业工作先进单位。切实维护农民工的合法权益，共清欠农民工工资9584万元，项目建设做到依法征地和拆迁，妥善解决好征地拆迁户的补偿和安置问题，保障群众的合法权益。维护了中国－东盟博览会、首届梧州国际宝石节期间的社会治安稳定。积极预防和化解人民内部矛盾，坚持“严打”斗争，加大综合治理力度，集中开展“命案必破”和打击“双抢一盗”、“地下六合彩”等专项斗争，加强网吧专项整治工作，并取得良好效果。全市基本实现“大事不出，中事不出，小事少出”的维护社会稳定的工作目标。高度重视安全生产工作，进一步完善和落实安全生产责任制，全市无重大责任事故发生。

上述成绩的取得来之不易，这是全市上下认真贯彻党的十六大和十六届三中、四中全会精神，真抓实干、艰苦奋斗的结果，也充分证明市人民政府贯彻上级的方针政策和市委的决策所作出的工作部署和安排是正确的，所采取的措施是切合实际、富有成效的。在此，我代表市人民政府，向全市人民，向驻梧部队官兵，向各民主党派、工商联、人民团体以及所有关心支持我市改革开放和现代化建设事业的各界朋友，致以衷心的感谢和崇高的敬意！

在充分肯定成绩的同时，我们也清醒地看到，目前我市经济社会发展中仍存在不少的困难和问题：一是农业生产基础设施还比较薄弱，抵御旱涝灾害能力不强，农民增收的基础不牢固；二是煤电油运供求形势依然严峻，严重制约我市经济发展；三是规模以上工业企业整体效益虽有明显好转，但亏损企业亏损额还比较大；四是财政收支矛盾突出，财政风险进一步加大；五是招商引资面临困难较多，合同履约率不高；六是低收入群体生活、下岗职工再就业、群众利益保护、三大纠纷调处等问题未得到很好解决。对这些困难和问题，政府将采取针对性措施，逐步加以解决。

二、2005年主要任务

2005年是承前启后全面实现“十五”计划目标、衔接“十一五”发展，实现我市经济和社会提速发展十分关键的一年。从当前形势看，做好我市今年的各项工作，虽然面临不少的困难和问题，但发展的环境和条件总体上是好的，可以说是机遇与挑战并存，但机遇大于挑战。因此，今年我市的各项工作仍然要以加快发展为第一要务，能快就不要慢，努力实现经济社会的提速发展。

今年政府工作的总体思路是：以邓小平理论和“三个代表”重要思想为指导，认真贯彻落实党的十六大、十六届四中全会和中央、自治区经济工作会议，以及市委十届十一次全会精神，坚持以科学发展观统领经济工作全局，抓住机遇，继续实施开放带动和项目带动两大战略，促进农业、工业交通、商贸旅游“三个轮子”快速驱动，千方百计在经济结构调整中增加经济总量，转变经济增长方式，提高经济运行质量，不断提高城乡居民生活水平，充分调动各方面积极性，实现经济社会全面协调可持续发展。据此，2005年我市经济和社会发展的主要预期目标初步安排为：

——生产总值增长12％。

——规模以上工业增加值增长18％。

——固定资产投资增长20％。

——财政收入增长12％。

——外贸出口增长11％。

——实际利用外资增长18％。

——社会消费品零售总额增长12％。

——城镇居民人均可支配收入增长8.5％；农民人均纯收入增长7％。

——城镇登记失业率控制在4.5％以内。

——人口自然增长率控制在7.8‰以内。

以上目标是我市经济和社会发展的最基本要求，体现了实事求是和积极稳妥的精神，只要我们认真贯彻落实中央和自治区的有关方针政策，齐心协力，埋头苦干，抓住时机，乘势而上，实现我市经济和社会各项事业更快更好地发展是完全有可能的。

今年要着力抓好以下几个方面工作：

（一）重视和加强“三农”工作，促进粮食增产和农民增收

全面落实科学发展观，坚持统筹城乡发展的方略，坚持“多予少取放活”的方针，把发展县域经济与加强“三农”工作有机地结合起来，与实施五大措施结合起来，进一步完善、强化和落实各项支农措施，保持农业和农村经济发展的好势头。

切实把解决好“三农”问题作为经济工作的重中之重，任何时候都不能放松。一是大力发展农业产业化经营，推进农业结构调整。正确处理好稳定粮食生产和发展多种经营的关系，今年粮食种植面积要达243万亩，总产量要达90万吨，确保全市粮食安全。在此基础上，加快培育一批能够带动广大农民增收致富的优势产业，积极抓好草食动物、瘦肉型猪、斑点叉尾鱼回等优质畜牧水产品种的推广和发展，大力发展速生丰产林，促使我市农业优势产业上规模、出效益。二是加强农业和农村基础设施建设。除继续争取国家、自治区各项支农资金，引导社会资金投资农业外，今年市财政将投入2470万元支持农业发展，比上年增长112.7％。三是深化农村各项改革，落实各项支农政策。全面推进农村税费改革，进一步降低农业税税率。积极稳妥地搞好乡镇机构改革、农村义务教育体制改革。继续抓好农村扶贫开发工作。依法稳定农村土地承包关系。按照自愿和互利互惠的原则，发展和壮大各种形式的农民专业合作经济组织。四是加大农业招商引资力度，积极引进和扶持能带动农民致富的农业龙头企业在我市落户、发展。

继续实施五大措施，做到力度不减，措施加强，质量提高，确保五大措施发挥应有的作用，实现预期目标。企业联动方面， 重点扶持15个农产品加工龙头企业，通过政府贷款贴息、用电用地等方面的倾斜，强化对企业任务的考核奖惩办法，促使其与基地建设、农户联合起来，真正成为带动农业产业化发展，促进农民增收的龙头企业。专业村带动方面，今年建设专业村100个，并逐步培育专业镇。突出抓好优势特色农产品规模化、标准化生产，力争新建成3个以上无公害农产品生产基地，并形成品牌产品。建立完善农产品质量安全检测体系，确保我市农产品的质量安全。劳务输出驱动方面，把提高农民工培训质量摆在工作首位，实施订单培训，保证培训的师资、内容、时间，使农民工掌握一门以上技术或技能，在满足市内企业用工需要的同时，抓好对外劳务输出，确保农民工培训和转移就业分别达6万人。销售队伍拉动方面，继续培育发展壮大80个销售大户，组建发展销售协会20个，加大扶持力度，力争今年的销售额增长28％以上。基础设施推动方面，重点抓好乡村公路和农田水利建设，计划安排农村基础设施项目162项，总投资1.6亿元，其中乡村公路75条，总里程420公里，水利项目62项，以带动我市农村基础设施上新台阶。

加大农村“三大纠纷”调处力度，维护农村社会稳定。“三大纠纷”是当前影响城乡社会稳定的重要因素之一。各级政府要按照属地管理、分级管理、分级办案和“谁主管、谁负责”的原则，妥善处理好“三大纠纷”。对未办结案件进行一次排查，对重大案件逐一登记造册并落实专人负责，年内新发生的案件要全部调结，历年积案调结率要达到80％以上，并建立群众越级上访处置机制，努力做好稳定工作。

（二）做大做强重点产业，全力加快工业发展步伐

贯彻落实《关于进一步加快工业发展的意见》精神，做大一批带动能力强、发展潜力大、市场前景好的企业。进一步加大协调力度，解决好煤电油运紧张问题，确保重点企业生产需要。突出发展民营经济，重点抓好林产林化、医药、日用化工、食品、宝石加工等五大工业产业，确保今年规模以上工业增加值完成预期目标，培育形成年销售收入超亿元的企业25家，其中1至2亿元的13个，2至5亿元的8个，5至10亿元的4家，形成骨干企业支撑产业发展的新格局，努力做大工业总量。争取有一批产品或品牌获得国家和自治区名牌产品或驰名商标、著名商标称号。

加快以工业园区为重点的工业载体建设。今年是工业园区的建设年、招商年，园区管委会要进一步完善园区的详规，加快园区建设步伐，加大项目引进力度，依法完成新征土地2300亩。全力推进“一园区多基地”建设，将龙圩、塘源基地纳入工业园区管委会统一管理。进一步理顺园区管理体制，建立灵活的用人机制，发挥园区的融资平台作用，努力把工业园区建设成为适应市场经济要求的经济实体。加强对进园项目的管理，提高入园项目土地单位面积投资额，防止圈地现象发生。对已签订合同但未按要求进资的项目用地要进行置换。进一步提升园区的服务质量，建立“一站式”服务体系，推行企业所有申报手续代办机制，增强园区的竞争力。2005年，旺甫基地已征土地要基本布满项目，并完成工业固定资产投资3亿元以上，力争年底前实现天盛纸业、荒川松香深加工、奥奇丽、中恒制药等9个项目建成投产。

深化和完善国有企业改革，完成改制扫尾工作。继续抓好尚未完成改制的国有企业的改制工作，积极争取金融部门的支持，妥善处置企业债务，依法实事求是地评估国有资产的价值，合理确定转让价格，为企业改制创造良好的条件。已完成改制的企业要抓好改制的善后工作，完善有关手续，依法解决好职工的养老、失业和医疗保险等问题，维护职工的合法权益，做好改制后的各项服务工作。重视城镇集体企业的改革发展工作，按照国家政策规定，给予集体企业应有的待遇，支持企业转换机制，加快发展步伐。

加大工业投入，增强发展后劲。计划今年开工和续建工业项目76项，完成投资额23.5亿元，其中工业基建项目完成16亿元，技术改造项目7.5亿元，完成工业前期工作项目16项，招商引资项目69项。

（三）多方筹措建设资金，推进三百项目大会战

主动适应国家加强宏观调控的新形势，把多方筹措资金、调整优化投资结构、推进重大项目建设更好地结合起来，保持投资对经济增长的强劲拉动。

今年三百项目大会战的主要安排是：建设项目130项，总投资约272亿元，年度投资约63亿元，其中新开工项目64个；前期工作项目73项，其中新开展的项目45项；招商项目100项，其中确定项目77项，外商自荐项目23项。为使三百项目建设卓有成效地开展，市政府将以筹措项目资金为工作核心，以工业项目为工作重点，以招商引资为突破口，突出抓好重大项目和市政建设项目，确保三百项目大会战顺利开展。

继续抓好重大项目建设。长洲水利枢纽要积极推进外江主体工程、外江船闸等工程建设，实现内江截流，完成投资15亿元；洛湛铁路要重点做好征地拆迁工作，支持工程建设，年内完成投资5亿元；苍郁高速公路要解决好与郁南接口问题，完成工程扫尾工作，实现通车；岑梧高速公路要加快施工进度，完成投资7亿元；梧州至马江高速公路要争取尽快开工。藤县至容县、黄村至昭平等5条二级公路要加快施工，确保任务完成。与此同时，抓好西江航道整治，提高西江干线航道等级，加快李家庄和新港港口建设，重振“黄金水道”雄风。扎实推进核电站、火电站、旺村水电站、肇庆至梧州铁路等一批重大项目的前期工作，增强我市经济提速发展的后劲。

从我市财力的实际出发，三百项目建设应主要靠市场运作推进。在大力推进银企合作、积极争取银行贷款的同时，要把工作重点放在招商引资和培育多元投资主体上，继续通过置换国有资产、盘活城市资产存量等方式，多渠道筹集建设资金。此外，继续实行三百项目目标责任制，确保各项目标任务的完成。

（四）以招商引资为重点，进一步扩大对外开放

进一步提高对招商引资重要性的认识，坚定“梧州经济发展的出路在招商”的信心。继续把粤港澳作为我市招商引资的主战场，逐步推进与长三角等区域的合作，推动梧州与江门、中山、肇庆等友好城市经贸合作协议的落实，力争与他们在梧州共建产业转移工业园，落实一批具体合作项目。积极向港澳台、日韩、东盟及欧盟等重点地区实行定点定向招商，力争利用外资有新的突破。办好第二届梧州国际宝石节，逐步完善政府推动、社会参与、市场运作的办节模式，进一步扩大宝石节的影响。

改进和完善招商引资工作机制和激励机制。进一步完善招商引资的政策措施，改善招商引资的软环境，调整招商引资的主体，改进考核、奖励的方法和内容，以实际到位资金作为考核奖励的唯一指标。同时，对去年合同履约情况进行一次检查，切实抓好签约项目的跟踪落实，提高合同履约率、项目开工率、资金到位率和投产率，进一步规范招商引资工作。

坚持以工业招商为重点，加大工业招商力度，努力提高第二产业在国民经济中的比重。在符合城市总体规划和土地利用规划的基础上，灵活安排工业项目用地，注重发展乡镇工业规划和建设，工业用地的出让价格，要按照国家有关规定来确定。同时，通过改善企业的基础设施来合理降低外商投资成本。坚持引进外资和内资一起抓，鼓励和引导社会投资，不断优化投资结构。

努力开拓国外市场，保持和发展对外贸易持续增长的良好势头。落实有关贸易便利化措施和国家出口退税政策，积极争取国家和自治区各种外贸发展专项资金，促进出口商品结构优化，帮助企业开展出口产品认证工作，推动优势产品创品牌，逐步提高出口产品竞争力。充分利用中国—东盟博览会和泛珠三角经贸合作区域建立的机遇，支持企业开展直接出口和境外加工贸易，促进我市进出口贸易的发展扩大。在抓好对外贸易的同时，加强各类专业市场建设，完善和优化现有市场，合理规划和建设商业网点，培育发展现代物流企业，进一步发展小城镇农贸市场，促进商贸流通业的大发展。

（五）加快城市建设步伐，确保“三年大变”目标的实现

2005年是我市实现城市面貌“三年大变”之年，一定要按质按量确保目标的实现。为此，今年市政建设的具体要求是，进一步拓展城市发展空间，加快红岭片规划和建设，做好南岸开发的前期工作。按照品牌路、样板路的要求，精心设计和建设好西堤路及各项配套设施，高质量、高品位、高速度建设沿路立面建筑，使之成为城市交通主干道和休闲观光旅游的新景点。加快市新博物馆和图书馆、西江明珠、长洲防洪堤、地质灾害整治等其他重点工程建设，积极推动火车站站前大道、红岭片开发、城市生活垃圾和污水处理等一批项目建设，完成城市建设投资9.13亿元。苍梧县龙圩镇的建设要纳入市区规划，统一管理。立足于招商引资，吸纳各类资金，参与城市建设，确保“三年大变”目标的实现。强化经营城市的理念，提高城市资源利用效益。继续开展建设用地清查，依法收回闲置土地。

切实加强城市管理，进一步加大市容环境的整治力度，逐步实施对小街小巷的改造，清理卫生死角，提高街道保洁度，创新路灯路树管理机制，改变园林建设滞后的状况，大力整顿车辆乱停乱放，树立环境优美管理有序的城市新形象。

认真贯彻落实我市《关于加快旅游产业发展的意见》，改革旅游管理体制，整顿和规范旅游市场秩序，改善旅游产业发展环境。切实抓好旅游发展规划，加大旅游资源开发和促销力度，做足“古”文章，重点抓好梧州龙母文化公园、四恩寺、珠山原英国领事署旧址等景区景点的建设和改造。整合全市旅游资源，重点支持蒙山太平天国封王遗址、藤县太平狮山、岑溪吉太漂流项目、苍梧飞龙湖休闲度假区开发，加强与周边城市的区域旅游合作，主动融入桂林、珠三角旅游圈。多渠道筹措资金，加快旅游业的发展。

（六）加强财税工作，确保财政金融平稳运行

培植财源与强化收入征管相结合，确保财政收入稳步增长。切实抓好财源培育工作，重点抓好财政对经济特别是对工业发展的支持，市财政安排2800万元，建立工业发展资金，支持企业做大做强。同时，多渠道筹措资金，安排4500万元支持工业园区建设，把工业园区建设成为财政持续增长的新亮点。加大对农业的投入力度，落实兑现五大措施的奖励资金。充分发挥工业发展资金作用，积极扶持和引导非公经济发展，支持外来投资者落户梧州，支持一批非公企业做大做强。

以增加财政收入为中心，依法强化税收征管。一是向管理要税收，向管理要效益，加强税收征管和监控，继续强化对房地产企业实行按预售收入预缴企业所得税的办法；二是抓好年纳税额10万元以上的税源培植，力争达到1000户；三是进一步盘活国有资产，实现国有资产收益1.18亿元以上；四是理顺市区两级财政管理体制，切实解决市和城区、城区与城区之间财政混库问题，制止互挖税源行为，充分调动市区两级组织收入的积极性。严格查处各种违法减免税行为，坚决制止和纠正擅自出台“先征后返还”等变相减免税政策，加强预算外收入的管理，挖掘非税收入潜力，确保财政收入稳步快速增长。

调整支出结构与加强支出管理相结合，确保重点支出需要。保证各项法定支出、工资发放和党政机关正常运转。加大就业、社会保障投入。压缩会议费、招待费等一般性支出，严格实行财政审批“一支笔”制度，严禁超预算、超财力开支。要以推进财政资金绩效评价为基础，强化资金跟踪问效，提高资金使用效益。

推动银企之间合作，大力发展地方金融，增强金融对经济建设的支持。支持金融改革，加强金融监管和金融服务。发展保险事业，为经济发展创造良好环境。规范社会信用秩序，努力培育良好的信用环境，发挥金融保险对地方经济和社会发展的推动作用。

（七）进一步壮大非公有制经济，使之成为我市经济的主力军

认真贯彻落实自治区党委、政府《关于加快民营经济发展的决定》，为非公有制经济发展创造平等竞争、一视同仁的法制环境、政策环境和市场环境。进一步放宽非公有资本进入基础设施、公用事业以及法律法规没有禁止的行业和领域。积极鼓励、支持和引导非公经济发展，落实扶持非公有制中小企业发展的投融资、财税信贷、技术创新等政策措施，加大金融、财政对非公经济的扶持力度，不断提高非公经济的产业层次和企业素质。对有条件的企业要帮助其上市直接融资。对非公企业申报符合国家产业政策的项目，有关部门要积极协助做好项目前期工作。进一步提高政府的工作效率和服务水平，加强与非公企业的联系，为其提供各种优质服务，维护企业合法权益，保护其正常经营活动。鼓励非公企业做大做强，重点培育红日公司、奥奇丽公司、广西荒川公司、蛋白肠衣厂、天盛纸业和百事达淀粉公司等一批非公有制集团企业，以壮大我市非公经济总量，促进我市经济快速发展。

（八）坚持实施科教兴市和可持续发展战略，大力推进社会各项事业的发展

高度重视科教事业发展。围绕加快工业发展的《意见》和农业“五大措施”，积极实施第三轮科技创新计划。突出抓好五大产业的技术创新和产品创新，解决好农业产业化、农产品增值等技术问题。继续抓好向农村选派科技特派员工作，带动农村科技进步。加大工业园区和农业实验区的科技扶持力度，提高两个园区的科技含量。继续巩固提高“两基”工作成果，完成8万平方米农村中小学危房改造任务。扩大普通高中办学规模，大力发展职业教育。在继续做好西大梧州分校申本工作，确保申本成功的同时，规划筹建梧州职业技术学院。鼓励和支持社会力量办学，完善多元办学体系。

努力发展文化、卫生、体育等其他各项事业。加强基层文化建设，抓好广播电视“村村通”工程，多渠道多形式丰富和活跃城乡居民的文化生活。加强文化市场建设和管理，实施艺术精品工程，加快发展文化产业。继续抓好新型农村合作医疗试点工作，全面推进乡村卫生服务一体化管理。进一步加强公共卫生体系建设，不断提高全市疾病预防控制能力和突发卫生事件应急处理能力。探索医疗服务体制改革，适度引入市场竞争机制。依法加大对非典、艾滋病等传染病和职业病防治工作。继续深入开展群众性体育活动，不断提高人民群众的身体素质。抓紧备战第十一届区运会，争创竞技体育新佳绩。抓好体训基地二期工程，力争开工建设市体育中心。加强多层次人才队伍建设，落实促进人才发展的各项政策措施，优化人才发展环境。加快实施事业单位人事制度改革。加强国防动员和民兵预备役工作，巩固和发展双拥工作成果。积极推进信息化建设，完善各级信息服务网络。做好防灾减灾工作。

坚持实施可持续发展战略，加强人口、资源和环境工作。对农村部分计划生育家庭实行奖励扶助制度，加强流动人口计划生育管理，稳定低生育水平，治理出生人口性别比偏高的问题，优化人口结构，提高人口素质。加强资源管理和环境保护工作。合理开发和节约使用资源，健全资源有偿使用制度，做好全市的节电节水工作。加强环境监督管理和污染治理，确保我市大气环境、地表水环境和城市噪声环境质量保持在优良状态。

（九）坚持以人为本，做好关系人民群众利益的各项工作

积极推进就业和再就业。认真落实各级目标责任，加大指导督查和考核力度，加快发展中小企业和劳动密集型产业，努力实现全年新增就业12000人，下岗失业人员再就业4000人的预期目标。同时，认真贯彻执行国家的劳动监察条例，依法规范劳资关系，严格执行政府颁布的最低工资标准，督促用人单位依法参加社会保险，保障职工的基本权益，提高就业和再就业的稳定性。切实把财政投入、税费减免、小额贷款、免费培训等扶持再就业的优惠政策落到实处，为下岗失业人员自主创业提供支持。建设和完善各级各类劳动力市场，加快推进下岗失业人员并轨工作，加强就业培训、职业介绍和就业服务指导，积极鼓励并大力发展灵活多样的就业形式。健全再就业援助制度，采取由政府购买公益性岗位的方式，优先安排“4050”人员就业，严格控制失业率。

努力推进社会保障体系建设。继续巩固“两个确保”，认真做好企业职工基本养老保险市级统筹工作，完善社会统筹与个人帐户相结合的基本养老保险制度。进一步扩大社会保障覆盖范围，提高非公有制企业的社会保险参保率。认真落实医疗保险政策，不断完善基本医疗保险体系。

重视解决困难群众的生活。建立健全城乡低收入困难群众社会救助体系，深入开展扶贫帮困活动，切实帮助城市下岗职工、烈军属、农村五保户、残疾人等弱势群体解决各种困难，妥善安排好他们的生活。城市居民最低生活保障工作做到应保尽保并逐步提高低保救助标准，使困难群众的基本生活得到保障。切实解决好拖欠农民工工资问题，依法保护农民工的利益。项目建设的征地拆迁补偿费要按国家政策规定的标准给予征地拆迁户，妥善安排好他们的生产生活。积极推进五保村建设，完善农村五保户生活保障制度。

（十）加强政府自身建设，提高经济社会管理水平

按照科学执政、民主执政、依法执政的要求，切实加强政府行政能力建设，努力建设开拓、务实、高效、廉洁的政府。

切实转变政府职能，建立服务型政府。加快政府由管理型向服务型转变步伐，把政府职能转到经济调节、市场监管、社会管理和公共服务上来。进一步减少和规范行政审批，提高政府机关的办事效率，全面推行政务公开，提高行政效能，提高政府公信力。强化社会管理，努力构建和谐社会。进一步加强对社会组织、社会事务的管理和规范，积极主动协调各种利益关系。加强法制宣传教育，做好信访工作，正确处理和妥善化解人民内部矛盾，维护社会政治稳定。完善社会治安防控体系，始终保持对刑事犯罪的高压态势，依法严厉打击各种违法犯罪活动，组织开展集中打击赌博违法犯罪活动专项行动，务必收到成效。建立健全各种突发事件应急机制，提高保障公共安全和处置突发事件的能力。高度重视和切实抓好安全生产，防止重特大安全事故发生。

坚持依法行政，努力建设法治政府。各级政府的领导干部要带头学法，增强有权必有责、用权受监督、侵权要赔偿的意识，提高依法行政水平和能力。加强公务员队伍建设，严格对执法人员的教育、管理和监督，努力提高他们的素质，切实做到严格执法、公正执法和文明执法。强化行政执法监督，认真解决有法不依、执法不公和多头执法、执法扰民等群众反映强烈的问题。进一步推进民主法制建设，各级政府要自觉接受同级人民代表大会及其常委会的监督和人民政协的民主监督，广泛听取社会各方面的意见。做好乡镇撤并工作，依法组织好全市村委会、社区居委会换届选举工作。

大力加强政风建设，进一步提高科学民主决策能力。一是积极开展保持共产党员先进性教育活动，牢固树立科学发展观，把维护好、实现好和发展好广大人民群众的根本利益作为政府一切决策的出发点和落脚点，真正做到权为民所用，情为民所系，利为民所谋。二是牢固树立正确的政绩观，做到各项决策的出台和实施都能经得起实践检验、群众检验和历史检验。各级政府作决策都要力求造福一方，坚决反对形式主义以及急功近利的行为。三是发扬民主，在决策过程中要广泛进行调查研究，倾听群众的呼声和意见，提高决策透明度和公众参与度。做好“十一五”规划的编写工作，提高规划的预见性、科学性和指导性。四是求真务实，切实转变工作作风。各级政府的领导要深入基层，帮助群众解决一些实际困难，解决重大问题。五是从严治政，保持清正廉洁。发扬艰苦奋斗的作风，增强拒腐防变能力，建立健全教育、制度、监督并重的惩治和预防腐败体系，严肃查处以权谋私、权钱交易等腐败案件，加大预防腐败工作力度，努力从源头上遏制腐败现象。

各位代表，2005年是承前启后的重要一年，做好今年的各项工作意义深远，责任重大。让我们紧密团结在以胡锦涛同志为总书记的党中央周围，高举邓小平理论和“三个代表”重要思想伟大旗帜，坚持科学发展观，在市委领导下，解放思想，抢抓机遇，开拓创新，扎实工作，为全面完成我市今年经济和社会发展的各项目标任务，实现梧州提速发展的目标而努力奋斗！
